# Immunoinformatics and reverse vaccinology approach in designing a novel highly immunogenic multivalent peptide-based vaccine against the human monkeypox virus

**DOI:** 10.3389/fmolb.2023.1295817

**Published:** 2023-11-22

**Authors:** Abhigyan Choudhury, Anshuman Chandra, Turki M. Dawoud, Hiba-Allah Nafidi, Nagendra Singh, Mohammed Bourhia

**Affiliations:** ^1^ Department of Animal Science, Kazi Nazrul University, Asansol, West Bengal, India; ^2^ School of Biotechnology, Gautam Buddha University (GBU), Greater Noida, Uttar Pradesh, India; ^3^ Department of Botany and Microbiology, College of Science, King Saud University, Riyadh, Saudi Arabia; ^4^ Department of Food Science, Faculty of Agricultural and Food Sciences, Laval University, Quebec, Canada; ^5^ Department of Chemistry and Biochemistry, Faculty of Medicine and Pharmacy, Ibn Zohr University, Agadir, Morocco

**Keywords:** monkeypox, TLR4, TLR2, molecular docking, reverse vaccinology, immunogenic, multi-epitope vaccine

## Abstract

**Background:** Monkeypox is a highly infectious zoonotic disease, often resulting in complications ranging from respiratory illnesses to vision loss. The escalating global incidence of its cases demands prompt attention, as the absence of a proven post-exposure treatment underscores the criticality of developing an effective vaccine.

**Methods:** Interactions of the viral proteins with TLR2 and TLR4 were investigated to assess their immunogenic potentials. Highly immunogenic proteins were selected and subjected to epitope mapping for identifying B-cell and MHC class I and II epitopes. Epitopes with high antigenicity were chosen, considering global population coverage. A multi-target, multi-epitope vaccine peptide was designed, incorporating a beta-defensin 2 adjuvant, B-cell epitopes, and MHC class I and II epitopes.

**Results:** The coordinate structure of the engineered vaccine was modeled and validated. In addition, its physicochemical properties, antigenicity, allergenicity, and virulence traits were evaluated. Molecular docking studies indicated strong interactions between the vaccine peptide and the TLR2 receptor. Furthermore, molecular dynamics simulations and immune simulation studies reflected its potent cytosolic stability and robust immune response dynamics induced by the vaccine.

**Conclusion:** This study explored an innovative structure-guided approach in the use of immunoinformatics and reverse vaccinology in pursuit of a novel multi-epitope vaccine against the highly immunogenic monkeypox viral proteins. The simulation studies indicated the engineered vaccine candidate to be promising in providing prophylaxis to the monkeypox virus; nevertheless, further *in vitro* and *in vivo* investigations are required to prove its efficacy.

## 1 Background

Monkeypox (MPX) is an infectious zoonotic disease caused by the enveloped double-stranded DNA (dsDNA) monkeypox virus belonging to the *Orthopoxvirus* genus within the Poxviridae family and represents an alarming health concern. While most cases present mild symptoms, certain populations, including children, pregnant women, and immunocompromised individuals, are at a high risk of severe complications from MPX, including bacterial infections, respiratory distress, and even vision loss (Bragazzi et al., 2023). The recent global surge in MPX infections, particularly in countries with no prior history of monkeypox, calls for urgent attention. As of August 2023, the World Health Organization (WHO) has reported a staggering 88,288 confirmed cases and even 149 deaths throughout the 112 countries affected (World Health Organization, 2023).

Some antiviral drugs that were originally devised against smallpox, such as tecovirimat, cidofovir, and brincidofovir, can also be repurposed against a few cases of MPX. Apart from conventional drugs, the development or repurposing of antimicrobial peptides against the disease is also a growing area of research. Some biocomputational studies postulate the inhibition of MPX infection or dissemination by the use of salivary histatin peptides and their autoproteolytic derivatives (Radhakuma et al., 2023) or by repurposing potential antiviral peptides against MPX (Miah et al., 2022; Singh et al., 2023). However, further *in vitro* and *in vivo* trials would be required to validate the claims. That said, there is currently no proven treatment for monkeypox (Das et al., 2023; Ghosh et al., 2023; Ivanov et al., 2023; Khani et al., 2023). Therefore, it is imperative to develop prudent prophylactic measures. A study based on data from a small African population indicates that smallpox vaccines can provide some amount of cross-protection; however, they lack specificity. Existing smallpox vaccines, such as ACAM2000, LC16, and MVA-BN (commercialized as Imvanex, Imvamune, or Jynneos), have been approved for active immunization against smallpox by the US Food and Drug Administration (FDA). That said, we must tread carefully as ACAM2000 is a live vaccinia virus-based preparation that carries the risk of secondary vaccinia infection and potential adverse effects like myocarditis or pericarditis in some recipients. While promising data from human immunogenicity trials and pre-clinical studies hint at the potential of MVA-BN to provide protection against MPX, a comprehensive large-scale clinical study is needed to determine its actual efficacy in safeguarding against monkeypox infection in humans. Similarly, LC16, a live, non-replicating attenuated vaccine approved for monkeypox prevention in Japan in August 2022, relies on extrapolated data from animal studies rather than direct human trials (Letafati and Sakhavarz, 2023; Reina and Iglesias, 2023; Srivastava et al., 2023; Xu et al., 2023).

**FIGURE 1:**
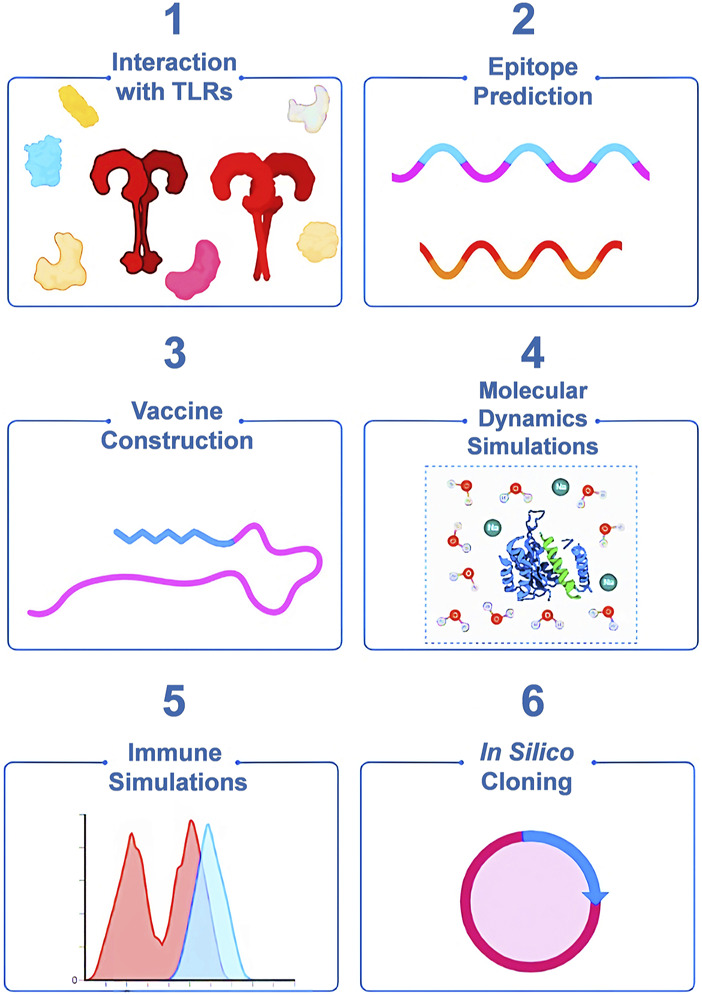
Generalized workflow of our structure-guided design of the highly immunogenic multi-epitope multivalent vaccine against MPX.

Given the increasing global burden of MPX infections and the limitations in treatment options, it is crucial to prioritize research and development efforts in the field of monkeypox vaccines. In recent years, by leveraging immunoinformatics techniques, multi-epitope vaccinology approaches have delivered strongly protective vaccine candidates against microbial infections while minimizing side effects compared to traditional vaccines (Gupta et al., 2016; Nagpal et al., 2018). In a novel approach presented in this study, we methodically target only those viral proteins that possess high immunogenicity and physiological reactivity to immune receptors. The highly immunogenic MPX proteins are further screened for epitopes with superior antigenicity. Subsequently, these epitopes are then used in the construction of the peptide vaccine structure (Figure 1). Peptide vaccination strategies represent a modern and time-tested vaccination platform that is being used against a wide variety of diseases, ranging from Alzheimer’s disease, HIV, malaria, and COVID-19 to cancer. In contrast to other modern vaccination platforms like mRNA vaccines, peptide vaccinations are highly scalable and economical as they are produced fully by chemical synthesis. Furthermore, these vaccines lend themselves to rigorous quality control assessments by established analytical methods like liquid chromatography and mass spectrometry, thereby ensuring high purity. Most importantly, the formulations can be stored and freeze-dried and do not require cold-chain facilities in storage, transport, or distribution, hence making it possible to vaccinate people even in the most remote corners of the world (Purcell et al., 2007; Dhanda et al., 2017; Usmani et al., 2017; Nagpal et al., 2018; Usmani et al., 2018; Malonis et al., 2020). Essentially, by utilizing this peptide vaccine, we hope to offer enhanced prevention and control over the monkeypox disease.

## 2 Significance of the study

The structural basis of immunogenicity of the MPX viral proteins remains virtually unexplored. The present study not only intends to bridge this gap but also harnesses the immunogenicity of the proteins to design a vaccination strategy against the disease.

## 3 Materials and methods

### 3.1 Structure retrieval of viral proteins

Every protein of the monkeypox virus is not involved in infection of the host, nor do they possess equal immunogenic capacities. Therefore, extensive literature research was carried out on the presently available proteomic data of MPX virions, using filters to screen the protein’s role in viral invasion pathways (Hatmal et al., 2022; Molteni et al., 2023) and its survivability. The in-depth research resulted in the derivation of 12 different viral proteins, and the primary structural data of the proteins were retrieved from the GenBank database (Benson et al., 2018). Cell surface-binding protein E8L (Accession ID: USO09213.1) is a transmembrane protein that possesses binding motifs for gangliosides on the lipid rafts, binds to the host cell receptor, and mediates viral entry into the cell (Fantini et al., 2022). Thymidine kinase (NP_536513.1) is important for replication and virulence of the virus and is an attractive antiviral drug target (Abdizadeh, 2023; Patel et al., 2023). DNA-dependent RNA polymerase (DdRp) subunit rpo132 (NP_536562.1) is essential for viral replication and is a promising drug target (Abduljalil et al., 2023), whereas profilin (USO09267.1) is involved in actin remodeling and plays an important role in the viral replication cycle (Minasov et al., 2022). Complement-binding protein (QNI40442.1) enhances viral survival by deactivating complement-based immune response from the host (Shchelkunov et al., 2002; Uvarova et al., 2005), and 36 kDa major membrane protein (QNI40458.1) is important in viral assembly and release (Sarkar et al., 2023). Poly A polymerase large subunit (QNI40472.1) plays a role in viral replication (Shchelkunov et al., 2002), E11L (AAL40566.1) is important for viral transcription, E13L (AAL40568.1) is a scaffold protein that gives immature virions a rigid and curved convex membrane (Farzan et al., 2023), and H6R (AAL40554.1) is a topoisomerase enzyme necessary for early transcription. Likewise, E6R (AAL40561.1) is required for virion assembly (Van Vliet et al., 2009), E4R (AAL40559.1) is a uracil DNA glycosylase and is functional in viral replication (Kannan et al., 2022), and E3R (AAL40558.1) is a structural protein and gives shape to the virion (Shchelkunov et al., 2002). Furthermore, using the SWISS-MODEL (Waterhouse et al., 2018) server from ExPASy, homology modeling methods were used to build coordinate structures of the abovementioned proteins. The stereochemical quality of these models was, nevertheless, validated using the structural assessment tool from ExPASy (Kiefer et al., 2009).

### 3.2 Interaction analyses of viral proteins to TLRs

In the present approach, we intend to design a novel vaccine candidate targeting only the highly immunogenic proteins of MPX. Studies have evidenced the role of TLR2 and TLR4 innate immune receptors in being the primal sensors for the viral proteins (Zhu et al., 2007; Van Vliet et al., 2009; Olejnik et al., 2018; Kaur et al., 2019; Sartorius et al., 2021; Zhou et al., 2021; Yu et al., 2021; Sanami et al., 2023; Tan et al., 2023). Hence, it was important to understand the interactions of the abovementioned proteins with the said TLRs to assess their immunogenic potencies. In this connection, structural data of TLR2–MD2 and TLR4–MD2 complexes were derived from the RCSB database (Rose et al., 2015) in the form of PDB ID: 6NIG and 3FXI, respectively. The coordinate data of the TLRs and MPX proteins were refined using PyMol (Yuan et al., 2017) and further used for intermolecular docking studies by using the ClusPro 2.0 supercomputer server (Kozakov et al., 2017; Alekseenko et al., 2020). The ClusPro server uses a PIPER algorithm that is based on the fast Fourier transform (FFT) correlation approach. It is a fast and accurate server that has always been a program of choice.

### 3.3 Screening of highly immunogenic proteins and epitope mapping

The designed vaccine candidate should be capable of generating a strong and durable immune response. In order to realize the same, we had to filter the most immunogenic MPX proteins; hence, we assessed the binding scores resulting from the molecular docking studies among the proteins and TLRs. The proteins showing the strongest fit with TLR2 and TLR4 were selected and used for the epitope prediction process. B-cell epitopes were extracted using the BepiPred-2.0 linear epitope prediction (Jespersen et al., 2017) algorithm offered by the Immune Epitope Database (IEDB). It is an enhanced sequence-based epitope prediction algorithm that can not only predict linear epitopes for a wide range of proteins with high accuracy but also analyze flexible regions that are quite challenging for other programs. An even and robust immune stimulation to T cell has been yet another pre-requisite; therefore, MHC class I and II epitopes were retrieved from the proteins using the T-cell epitope prediction and analysis tools from IEDB. We used NetMHCpan 4.1 EL and NetMHCIIpan 4.1 EL for MHC class I and II epitopes, respectively (Trolle et al., 2015; Andreatta et al., 2018). These algorithms are precise and versatile, supporting high-throughput data handling. For maximizing the coverage for more than 95% of the world population, specific MHC-I alleles were chosen: HLA-A*02:01,9, HLA-B*15:01,9, HLA-A*02:06,10, HLA-A*03:01,9, HLA-B*54:01,10, and HLA-B*51:02,9. Similarly, MHC-II alleles of H2-IAb, HLA-DPA1*01/DPB1*04:01, HLA-DRB1*01:01, HLA-DRB1*13:01, HLA-DRB1*03:01, HLA-DRB1*04:01, HLA-DRB1*07:01, HLA-DRB3*01:01, HLA-DRB1*08:01, HLA-DRB1*11:01, HLA-DRB3*02:02, HLA-DRB5*01:01, HLA-DRB4*01:01, and HLA-DRB1*15:01 were used (Solanki and Tiwari, 2018). Furthermore, the top 0.2 percentile epitopes were selected as they were ranked by their binding affinity toward the MHC proteins. VaxiJen 2.0 is a server based on the transformation of protein sequence auto cross-covariance (ACC) into uniform vectors of main amino acid properties (Doytchinova and Flower, 2007; Zaharieva et al., 2017; Salod and Mahomed, 2022). It was used to determine the antigenicity of the derived epitopes against a virus model, and only the high-ranking epitopes were picked.

### 3.4 Structural construction and validation of the vaccine candidate

A vaccine design must support the proper folding and presentation of antigenic peptides, and it must enable epitopes to be accessible to the immune cells while also stabilizing the protein structure of the vaccine. Hence, it is necessary to use linkers for joining the epitopes in the structure. The primary structure of the construct peptide initiated with a highly immunogenic adjuvant of beta-defensin 2 (Accession ID AAC69554.1) (García-Valtanen et al., 2014; Kim et al., 2018) was linked to a series of MHC-I epitopes using an EAAAK linker. The MHC-I epitopes, however, were connected to each other using AAY linkers. The B-cell epitopes were joined to each other using the KK linkers. Similarly, MHC class II epitopes were linked using GPGPG sequences. Thereafter, the tertiary structure of the multi-target multi-epitope vaccine peptide was generated by the transform-restrained Rosetta (trRosetta) server that uses deep learning methods for highly accurate *de novo* protein modeling (Du et al., 2021). The model was subjected to stereochemical validation using the ERRAT program provided by the SAVES 6.0 server (Colovos and Yeates, 1993); it is a highly sensitive program that verifies protein structures based on the statistics of non-bonded atom–atom interactions in the structure and compares it with a database of reliable high-resolution structures. Furthermore, the SWISS-Model Structural Assessment tool was also used for the purpose.

### 3.5 Physiochemical evaluation of the vaccine peptide

Following the design of the vaccine, it is imperative to assess its different physiochemical properties. ExPASy ProtParam (Garg et al., 2016) was primarily used for analyzing the physiochemical characteristics of the vaccine peptide. Furthermore, its overall antigenicity was determined using the VaxiJen 2.0 server. The allergenic nature of the peptide was assessed using AllerTOP 2.0 (Dimitrov et al., 2013; Dimitrov et al., 2014), and VirulentPred (Garg and Gupta, 2008) was used for the evaluation of the virulence traits of the vaccine. Moreover, the ElliPro (Ponomarenko et al., 2008; Usmani et al., 2018) tool from IEDB was used to map the conformational B-cell epitopes of the vaccine. It is known for its accuracy in predicting the discontinuous epitopes based on the special positioning of residues in the protein.

### 3.6 Molecular docking with TLR

To gain insights into the immunological instigatory capacities of the vaccine, the peptide was docked to the TLR2 receptor using the ClusPro 2.0 supercomputer server, and the interactions were studied using PyMol.

### 3.7 Molecular dynamics simulations and binding energy

Inspecting the binding interactions of the vaccine and the receptor TLR2 demands their dynamic behavior to be kept in consideration, and thus, the molecular dynamics (MD) simulations are performed. For a comprehensive analysis, the TLR2 protein was simulated primarily in its apo form, and thereafter, the vaccine-TLR2 complex was operated by using the Desmond module from the Schrodinger suite. The proteins were immersed in a TIP3P predefined water solvent model delimited by orthorhombic periodic boundary conditions. Furthermore, electrical neutralization was achieved by adding appropriate amounts of sodium and chlorine ions, whereby the system was minimized under the OPLS3e force field (Roos et al., 2019). The NPT ensemble (Kim et al., 2019) was used to run the simulation at a constant temperature of 300 K at 1 atm pressure for 100 ns (Basconi and Shirts, 2013). The stability of the proteins was analyzed in terms of different parameters, like root mean square deviation (RMSD), root mean square fluctuation (RMSF), the radius of gyration (Rg), and solvent-accessible surface area (SASA). Moreover, molecular mechanics with generalized Born surface area (MM-GBSA) calculations were performed in order to evaluate the overall binding strength of the vaccine–receptor complex.

### 3.8 Immune simulation studies

By modeling the interactions between the vaccine antigens and immune cells, immune simulations can serve as a cost-effective and time-efficient approach for predicting the magnitude and kinetics of the immune response induced by the designed vaccine. The C-ImmSim server provides an agent-based model that uses position-specific scoring matrix (PSSM) scores derived from machine learning techniques for predicting immune interactions (Castiglione et al., 2007; Rapin et al., 2011). Employing the server, a 35-day-long simulation was run, and a dose of the vaccine peptide was injected on the very first day. The subsequent immune response dynamics were noted.

### 3.9 Reverse translation and *in silico* cloning

Efficient and fast manufacturing of the vaccine candidate poses a significant step toward herd immunity, one that is achieved through cloning the vaccine codons into highly optimized expression vectors. Hence, the vaccine peptide sequence was first reverse-translated through the *E. coli* K12 codon table using EMBOSS Backtranseq (Rice et al., 2000), and SnapGene ver.6.1 software was used to clone it into a pET-28a (+) expression plasmid vector (Mauro and Chappell, 2014).

## 4 Results

### 4.1 Immunostimulatory potency of viral proteins

**FIGURE 2:**
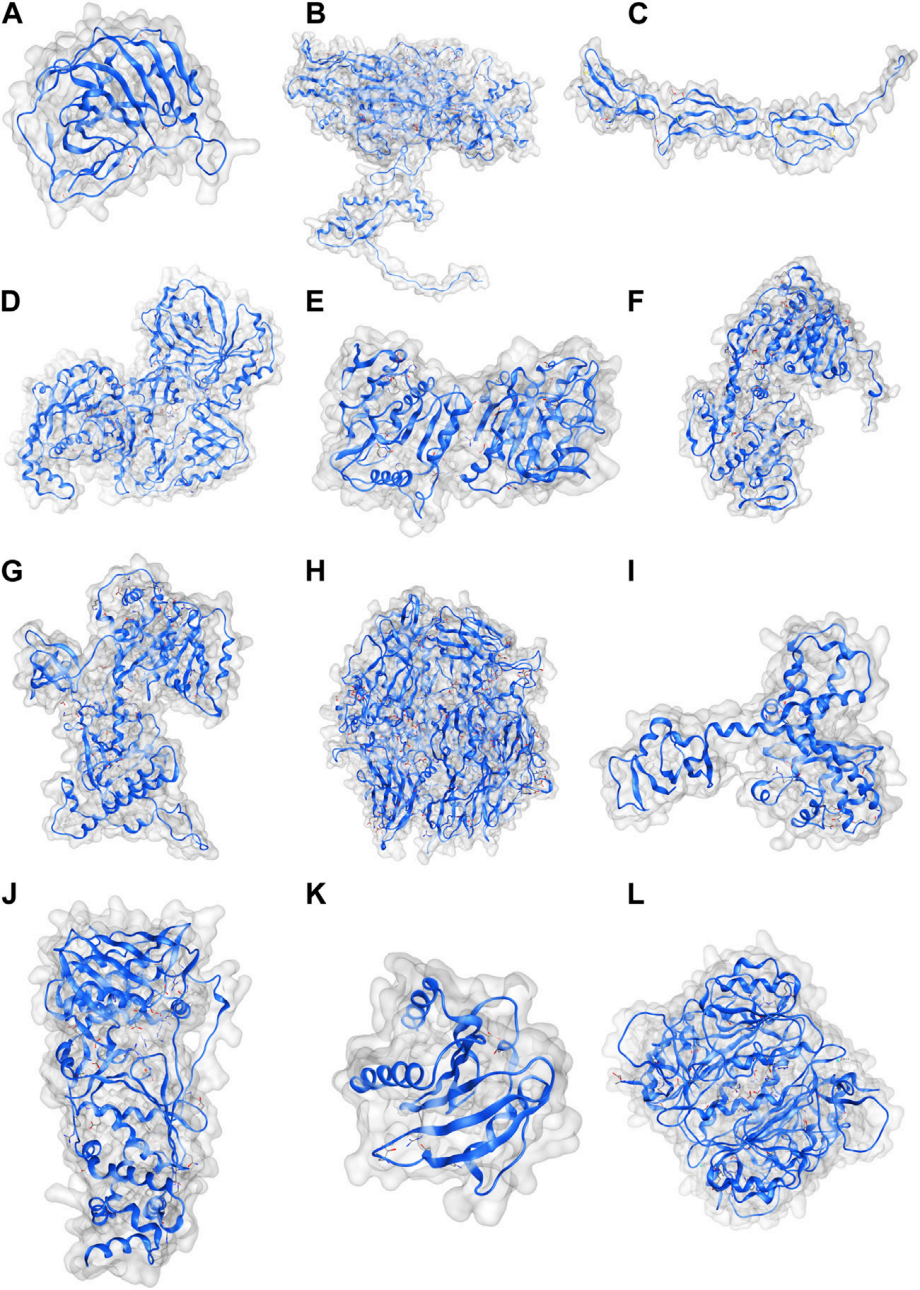
Coordinate structures for MPX viral proteins: **(A)** Cell surface-binding protein, **(B)** DNA-dependent RNA polymerase subunit rpo132, **(C)** complement-binding protein, **(D)** E3R, **(E)** E4R, **(F)** E6R, **(G)** E11L, **(H)** E13L, **(I)** H6R, **(J)** poly-A polymerase large subunit, **(K)** profilin, and **(L)** thymidine kinase.

**FIGURE 3:**
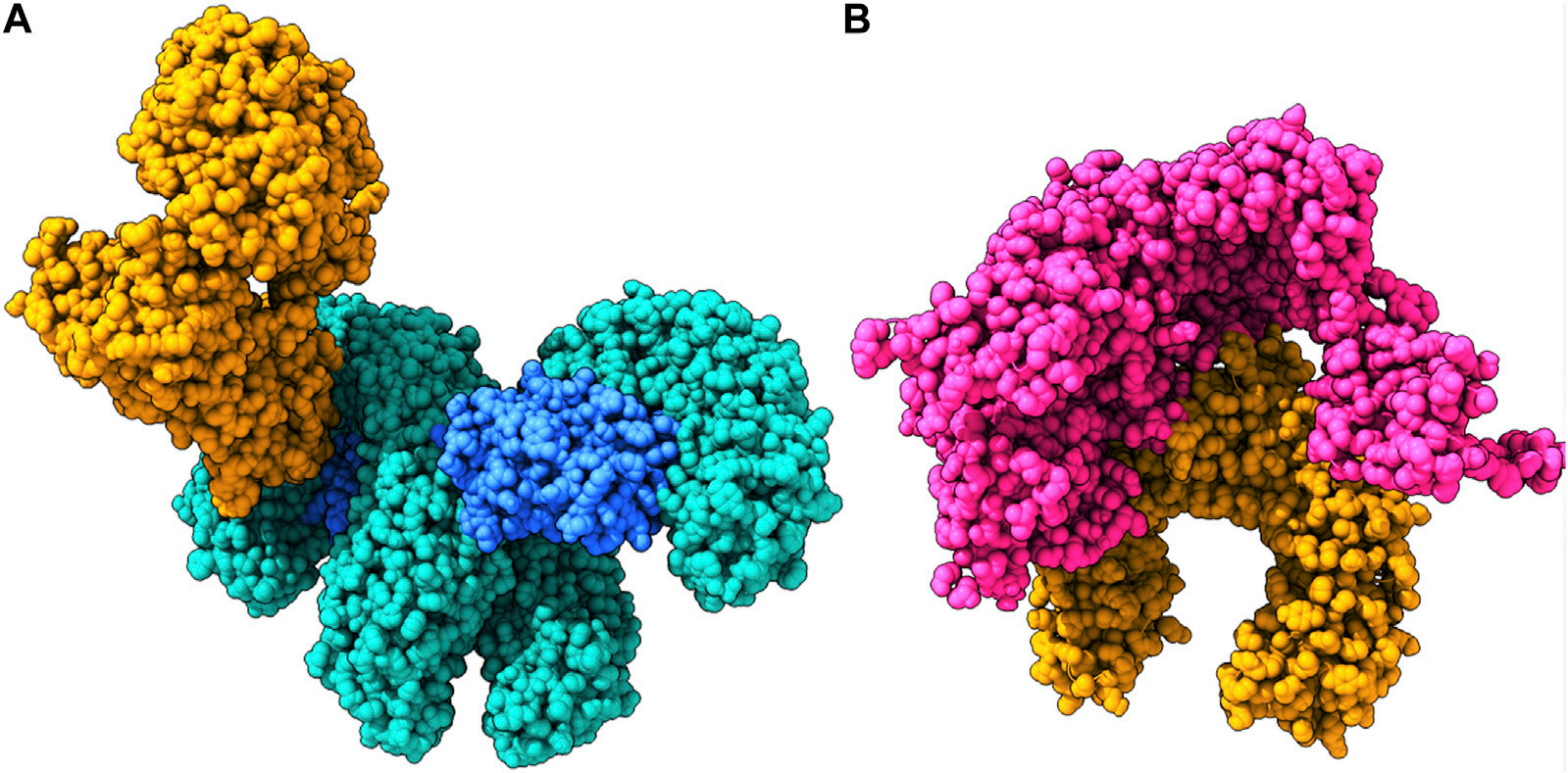
**(A)** E6R protein bound to the TLR4–MD2 complex. **(B)** DNA-dependent rna polymerase subunit Rpo132 bound to the TLR2 receptor.

Coordinate structures of the MPX proteins were prepared by homology modeling (Figure 2), followed by their structural assessment, which revealed that all the structures were quite precise naturally and had 92.63%–98.71% residues in the Ramachandran-favored regions, as visualized in the plots of Supplementary Figure S1. Furthermore, they were subjected to molecular docking studies against TLR2 and TLR4 to understand the immunostimulatory abilities of the proteins. The studies reflected that except for the complement-binding protein, all the MPX proteins were able to bind TLR2, and the strongest interactions were found in the case of the DdRp subunit rpo132, scoring −1327.6 at ClusPro (Figure 3B). Similarly, TLR4 was able to detect all the proteins; however, exceptionally well results were observed with the E6R protein, scoring −992.9 (Figure 3A).

### 4.2 Prediction and screening of immunogenic epitopes

The immunogenic proteins of the DdRp subunit rpo132 and E6R were now scanned for different forms of epitopes present. The BepiPred-2.0 linear epitope prediction algorithm predicted 23 B-cell epitopes present in the E6R protein and 22 such epitopes in the DdRp subunit rpo132. MHC-I epitopes of the proteins were retrieved using the NetMHCpan EL4.1 (Reyn et al., 2020) algorithm against HLA-A*02:01,9, HLA-B*15:01,9, HLA-A*02:06,10, HLA-A*03:01,9, HLA-B*54:01,10, and HLA-B*51:02,9, thereby choosing epitopes from the 0.2 percentile of top-binding epitopes. Strongly binding MHC-II epitopes, on the other hand, were predicted using the NetMHCIIpan 4.1 EL method against the alleles of H2-IAb, HLA-DPA1*01/DPB1*04:01, HLA-DRB1*01:01, HLA-DRB1*13:01, HLA-DRB1*03:01, HLA-DRB1*04:01, HLA-DRB1*07:01, HLA-DRB3*01:01, HLA-DRB1*08:01, HLA-DRB1*11:01, HLA-DRB3*02:02, HLA-DRB5*01:01, HLA-DRB4*01:01, and HLA-DRB1*15:01. All predicted MHC-I and MHC-II epitopes were then subjected to antigenic evaluation using the VaxiJen 2.0 server. The filtration further resulted in 16 MHC-I and six MHC-II highly antigenic epitopes from the E6R protein, in addition to 18 MHC-I and five MHC-II antigenic epitopes present in the DdRp subunit rpo132. The epitopes were then ranked, and only the ones with the highest immunogenic values were chosen.

### 4.3 Designing of the multi-target, multi-epitope vaccine peptide

**FIGURE 4:**
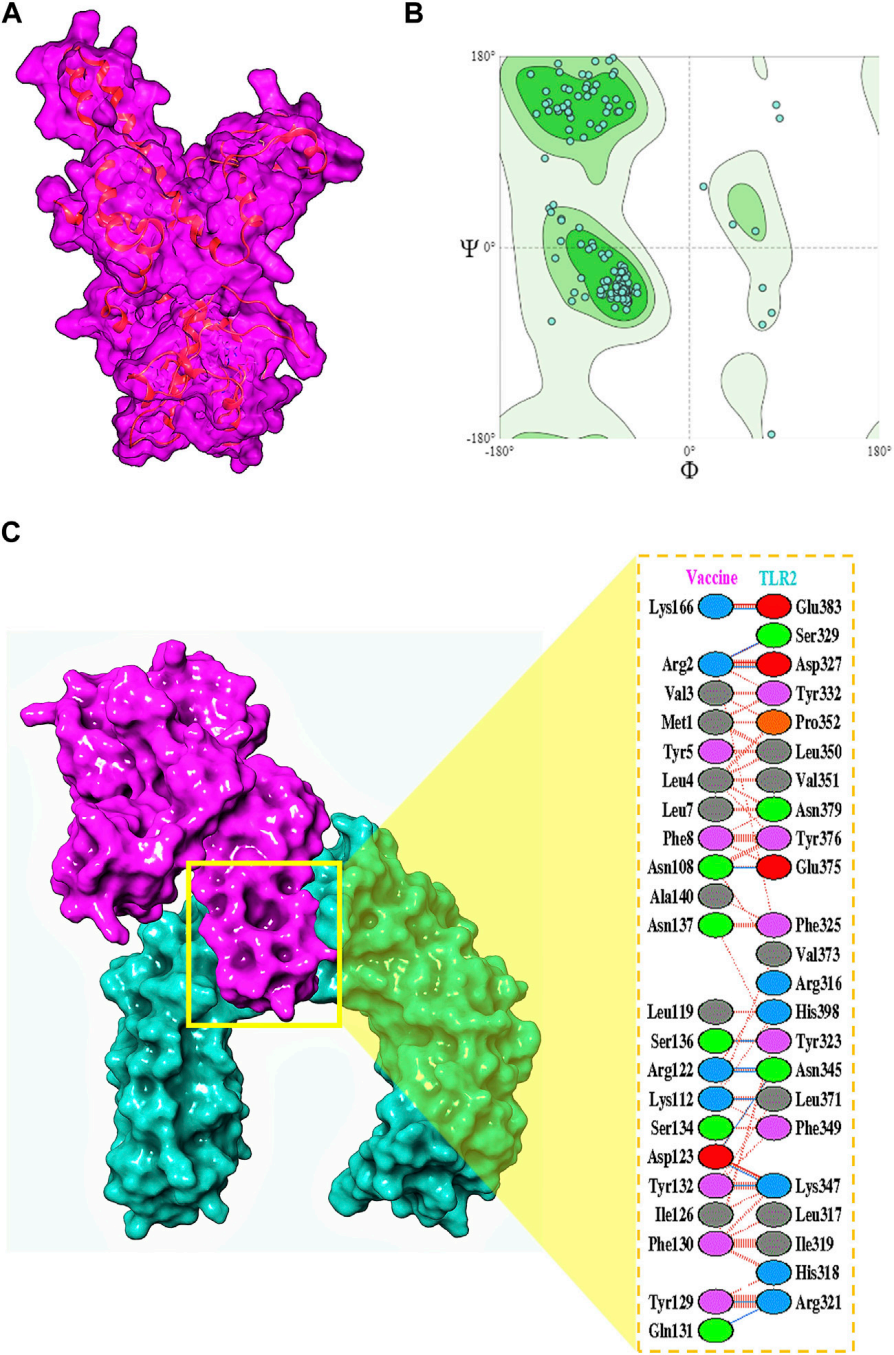
**(A)** Multivalent vaccine presented in its surface form, with its backbone visibly illustrated. **(B)** The Ramachandran plot for the vaccine. **(C)** Its interactions with the TLR2 receptor.

The final vaccine structure consisted of four main domains—the beta-defensin 2 adjuvant, the B-cell epitopes, the MHC-I epitopes, and the MHC-II epitopes. The tertiary model structure (Figure 4A) prepared by trRosetta by its *de novo* modeling algorithms was evaluated for its structural viability, and it was revealed to have a high ERRAT score of 95.8647 and composed of 94.75% Ramachandran-favored residues (Laskowski et al., 2013) according to the SWISS-Model Structural Assessment tool (Figure 4B).

### 4.4 Physiochemical characteristics of the vaccine

The vaccine candidate weighed 33.8 kDa, and its cytosolic half-life was found to be 30 h in mammalian reticulocytes, more than 20 h in yeast, and more than 10 h in *E. coli*. The vaccine was found to be stable, with an instability index of 22.72 and an aliphatic index of 94.07. Additionally, the GRAVY (grand average of hydropathy) value was reported to be −0.156. As reported by VaxiJen 2.0, the overall antigenicity of the vaccine was as high as 0.8197. Furthermore, it was found to be non-allergenic by AllerTOP and scored −1.024 in VirulentPred analysis, which is indicative of its avirulent nature. In addition, ElliPro revealed seven different conformational B-cell epitopes present in the vaccine structure (Supplementary Figure S2).

### 4.5 Interaction of the vaccine to immune receptors

To understand the biophysical basis of immunogenicity of the vaccine at the cytosolic level, the peptide was first docked to the surface immune receptor of the TLR2 receptor using ClusPro, and strong binding poses were determined, with the highest scoring being −1472.6. The interface of this complex was explored, which revealed the orchestration of the binding interactions by different residues from the TLR2 as well as the vaccine peptide (Figure 4C). Glu383, Ser329, Asp327, Tyr332, Pro352, Leu350, Val351, Asn379, Tyr376, Glu375, Phe325, Val373, Arg316, His398, Tyr323, Asn345, Leu371, Phe349, Lys347, Leu317, Ile319, His318, and Arg321 of TLR2 were found to offer binding interactions to the vaccine residues of Lys166, Arg2, Val3, Met1, Tyr5, Leu4, Leu7, Phe8, Asn108, Ala140, Asn137, Leu119, Ser136, Arg122, Lys112, Ser134, Asp123, Tyr132, Ile126, Phe130, Tyr129, and Gln131 (Table 1).

**TABLE 1:** Interaction strength of the vaccine peptide with the TLR2 receptor protein.

Immune receptor bound to the vaccine peptide	Interacting residues		Binding free energy (MM-GBSA)
	Immune receptor	Vaccine peptide	
**TLR2**	Glu383, Ser329, Asp327, Tyr332, Pro352, Leu350, Val351, Asn379, Tyr376, Glu375, Phe325, Val373, Arg316, His398, Tyr323, Asn345, Leu371, Phe349, Lys347, Leu317, Ile319, His318, and Arg321	Lys166, Arg2, Val3, Met1, Tyr5, Leu4, Leu7, Phe8, Asn108, Ala140, Asn137, Leu119, Ser136, Arg122, Lys112, Ser134, Asp123, Tyr132, Ile126, Phe130, Tyr129, and Gln131	−130.8138 kcal/mol

### 4.6 Molecular dynamics simulations and MM-GBSA calculations

Molecular dynamics simulations have proven to be instrumental in guiding the process of experimental validation. The simulation studies facilitate the examination of conformational alterations, stability fluctuations, and the holistic progression of complex systems when replicated under conditions mimicking the cellular cytosol environment. They enable the comprehensive assessment of critical parameters, such as RMSD, RMSF, Rg, and SASA. In this comprehensive study for determining the stability of the vaccine–TLR2 complex, we first simulated the native TLR2 receptor. Furthermore, under the same conditions, we simulated the evolution of the vaccine–TLR2 complex, consequently obtaining a comparative view.

**FIGURE 5:**
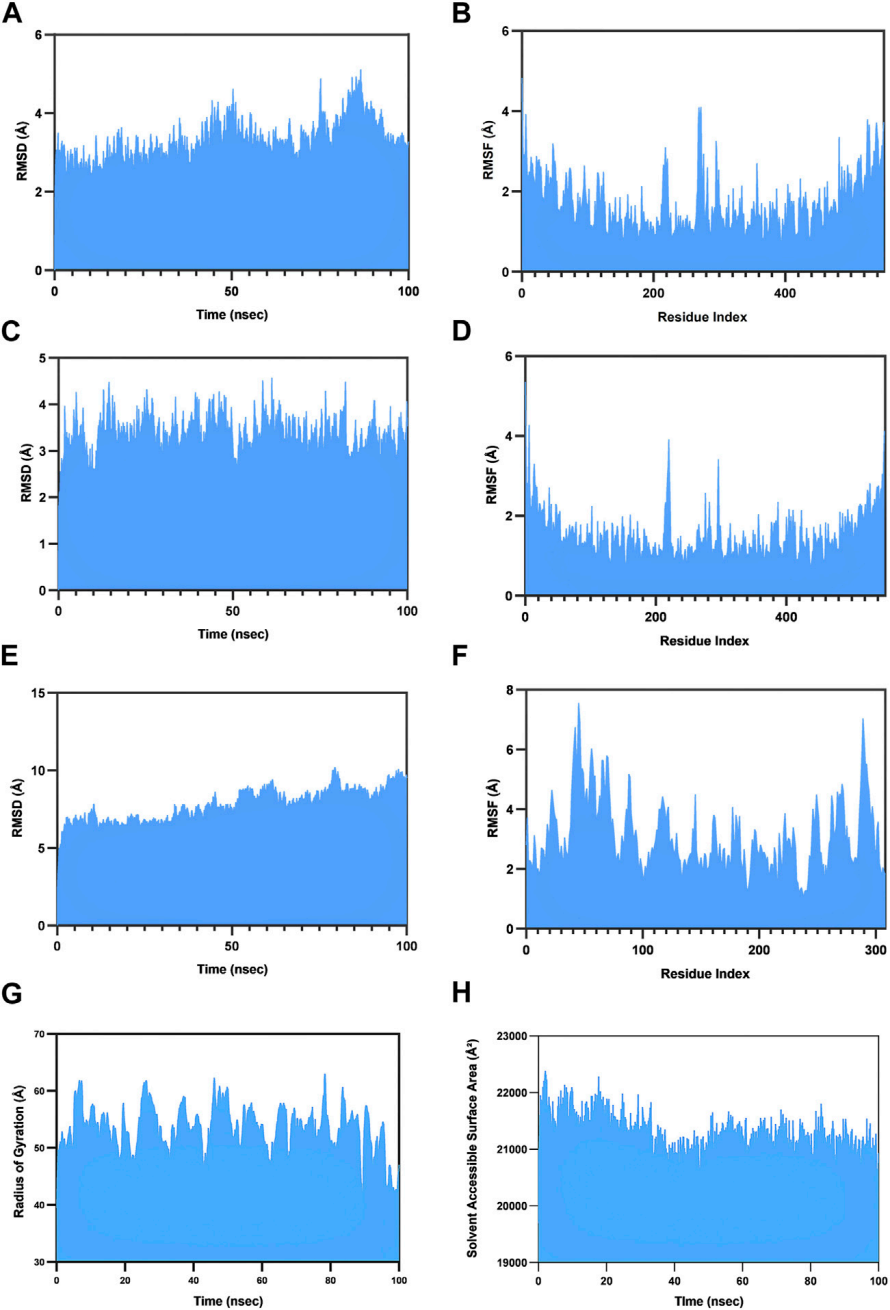
Molecular dynamics simulation studies. The dynamics of the natural (unbound) TLR2 receptor is exhibited in terms of **(A)** RMSD and **(B)** RMSF. This is compared with the dynamics of the TLR2 receptor when bound to the vaccine. **(C)**, **(D)** RMSD and RMSF, respectively. **(E)**, **(F)** Visualization of the same parameters for the bound vaccine. **(G)** The evolution of the radius of gyration (Rg) of the vaccine–TLR2 complex; **(H)** the same in terms of the solvent-accessible surface area.

RMSD stands as a pivotal quantifiable metric characterizing the stability of the protein structure. Elevated RMSD values correlate with diminished anticipated protein stability, whereas reduced values of RMSD are indicative of heightened stability. The native TLR2 structure has an average RMSD of 3.27 Å (Figure 5A); however, when bound to the vaccine construct, it exhibited a similar RMSD of 3.32 Å (Figure 5C). Interestingly, increased stability of the TLR2 in the bound pose is, essentially, perceived from the 70th ns to the 90th ns in the course of the simulation. The vaccine construct remained stable during the whole simulation and had an average RMSD of 7.75 Å (Figure 5E).

The RMSF parameter is commonly used in molecular dynamics simulations to investigate the flexibility and fluctuations of the protein backbone at the residue level. The simulation revealed that the TLR2 bound to the vaccine has a mean RMSF of 1.49 Å (Figure 5B), which is slightly lower than that of the native TLR2 having an average RMSF of 1.66 Å (Figure 5D). Similarly, the vaccine construct had a 3.06 Å mean RMSF (Figure 5F). These findings are indicative of the enhanced stability of the complex.

The observed stability of the complex can be elaborated by other different parameters as well*.* Rg defines the compactness of the complex. The average Rg of the vaccine–TLR2 complex was found to be 53.4 Å throughout the trajectory (Figure 5G). On the other hand, SASA measures the area of a protein’s surface that is accessible to solvent molecules. The complex has an average SASA of 21243.11 Å^2^, and simulation reveals a mild but gradual reduction in SASA until the 100th ns, further reflecting heightened stability (Figure 5H).

The calculation of binding free energy is a critical parameter in elucidating the molecular recognition process. We used the MM-GBSA for the purpose as it combines the classical force field method with the generalized Born continuum solvent model and offers us a good estimate of the binding energy. Between 50th ns to 100th ns of the simulation, the complex exhibited an average ΔG of −130.81 kcal/mol with minima and maxima of −72.94 and −178.28 kcal/mol, respectively.

The overall results of the MD simulations suggest that the vaccine construct forms stable and consistent complexes with immune receptors under the emulated microenvironmental conditions, indicating its strong immunostimulatory potency.

### 4.7 Prediction and analyses of the immunostimulatory dynamics

**FIGURE 6:**
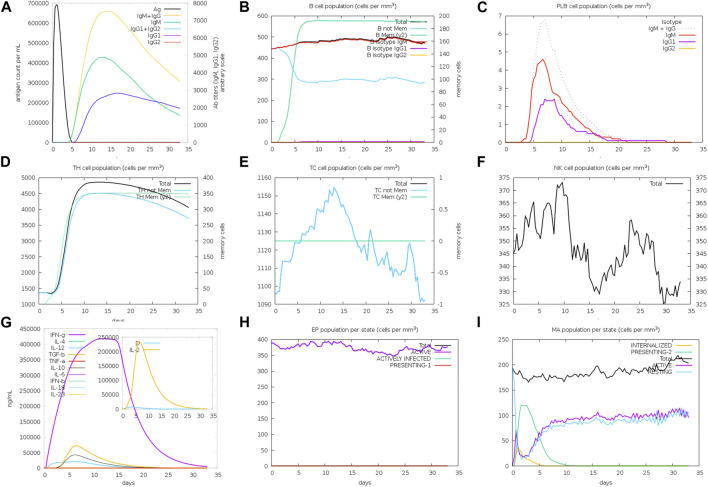
**(A)** Titers of immunoglobulins and the immunocomplexes after vaccination. **(B)** Levels of B-cell population after vaccination. **(C)** Levels of plasma B-cell after vaccination. **(D)** Levels of helper T-cell population after vaccination. **(E)** Levels of cytotoxic T-cell population after vaccination. **(F)** Levels of NK-cell population after vaccination. **(G)** Concentration of cytokines and interleukins after vaccination. **(H)** Epithelial cell levels. **(I)** Levels of MA population after vaccination.

In order to assess the effectiveness of a vaccine peptide, it is crucial to simulate the natural immune reactivity before proceeding to further trials. The C-ImmSim simulation platform that uses Miyazawa and Jernigan protein–protein potential measurements was used for this purpose. The simulation began by administering the vaccine dose in the first hour of a 35-day-long stimulation period (Figure 6). The immune response observed during this simulation demonstrated elevated levels of IgM and IgG antibodies, which together compose the primary immune response. Subsequently, there was a gradual increase in the concentrations of IgM, IgG1, and IgG2 antibodies, accompanied by a decrease in antigen titers. Moreover, we observed robust secretion of cytokines, specifically IFN-y and IL-2. These findings collectively indicate the superior immunogenic capacity of the novel vaccine peptide.

### 4.8 *In silico* cloning of the vaccine

**FIGURE 7:**
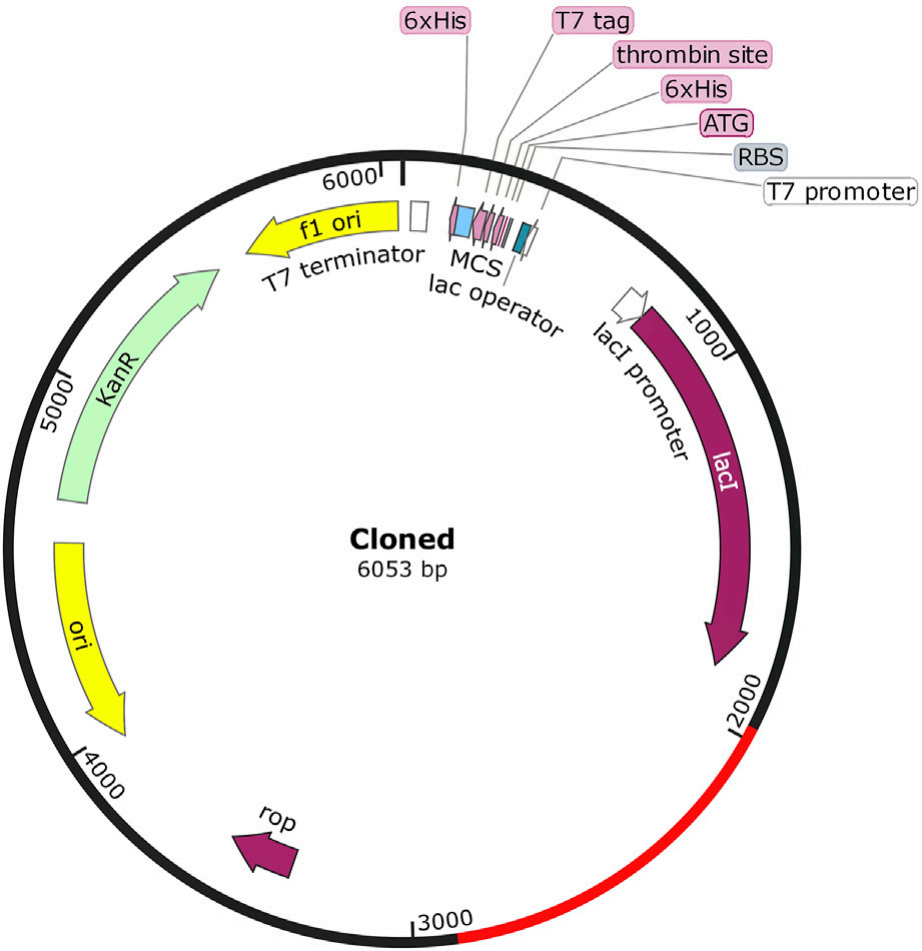
Map of the cloned pET-28a (+) plasmid vector, with the vaccine sequence highlighted in red.

Efficient and fast manufacturing of the vaccine candidate poses a significant step toward herd immunity, one that is achieved through cloning the vaccine codons into highly optimized expression vectors. Hence, the vaccine peptide sequence was first reverse-translated through the *E. coli* K12 codon table using EMBOSS Backtranseq, which revealed a transcript with a GC of 52.23. It indicates optimal processivity and expression in *E. coli* systems. Finally, the SnapGene ver.6.1 software was used to clone it into a pET-28a (+) expression plasmid vector (Figure 7).

## 5 Discussion

The preceding year has seen the monkeypox disease making a concerning shift from its usual patterns and spreading simultaneously across multiple countries (Islam et al., 2022). Ongoing research against the disease has claimed the possible efficacy of some conventional antiviral drugs, as well as therapeutic peptides. Unfortunately, the claims await validation, and as of now, no effective treatments or preventive measures are available for MPX infections, underscoring the urgent need for a competent vaccine. One promising avenue is the development of a multi-epitope vaccine based on reverse vaccinology. Some preliminary studies have attempted and proposed few designs of such vaccine candidates based upon an approach where the construct is composed of a few epitopes for several MPX proteins, resulting in the development of dispersed immune memory. Due to limitations of scope, however, the studies lacked any investigation into the structural basis of immunogenicity of the viral proteins, upon which the reverse vaccinology computation stands (Akhtar et al., 2022; Bhattacharya et al., 2022; Jahantigh et al., 2023; Rcheulishvili et al., 2023; Waqas et al., 2023). The design presented in this article, nonetheless, relies on proteins that can exhibit high immunogenicity and activate primary innate immune cascades. We examined the interactions between the viral proteins and TLR2 and TLR4. Molecular docking studies elucidated that TLR2 strongly recognizes the DdRp subunit Rpo132, while TLR4 is highly sensitive toward the E6R protein. Combining T-cell and B-cell epitopes in polypeptide-based vaccines has been shown to significantly enhance immunogenicity compared to using these epitopes individually. Hence, the DdRp subunit Rpo132 and E6R were analyzed for the prediction of B-cell as well as both forms of T-cell epitopes, i.e., MHC-I and MHC class II epitopes. Furthermore, only highly antigenic epitopes were selected after a wide screening process. Beta-defensin 2 was chosen as the primary adjuvant molecule, and the different components of the vaccine were amalgamated using EAAAK, AAY, KK, and GPGPG linkers. Upon analyzing the predicted biophysical and biochemical characteristics of the vaccine construct, it revealed promising attributes of antigenicity, safety, stability, thermostability, and hydrophilicity. The construct also demonstrated appropriate solubility upon expression. Certainly, allergies have been a concern regarding new vaccine candidates; however, our construct was evaluated to be non-allergenic. These encouraging findings designate it as an excellent vaccine candidate; hence, it was subjected to further in-depth analysis and evaluation. The molecular docking analysis indicated that the multi-epitope vaccine exhibits robust binding affinities with TLR2. This finding confirms the vaccine’s ability to be recognized by the innate immune pathways, leading to the induction of stable and potent immune responses. The stability of the vaccine–TLR2 complex under diverse microenvironmental conditions, encompassing pressure, temperature, and motion, was assessed through molecular dynamics simulation analysis. Initial trajectory evaluations, including calculations of RMSD, Rg, RMSF, and hydrogen bonds, consistently demonstrated the high stability of the vaccine–TLR2 complex. Additionally, the MM-GBSA calculations revealed strong negative values for the binding free energy, indicating the ability of the complex to maintain stability under natural conditions. In general, the processing and presentation of cytotoxic T-cell and helper T-lymphocyte epitopes occur through distinct pathways, specifically, proteasomal degradation (endogenous) for cytotoxic T-cell epitopes and endo-lysosomal degradation (exogenous) for helper T-lymphocyte epitopes. The multi-epitope vaccine is classified as an extracellular antigen. Once injected into the host body, the vaccine primarily triggers the activation of CD4^+^ T cells through the exogenous pathway, thereby inducing adaptive immunity. Subsequently, B cells are activated to generate humoral immunity. In the realm of synthetic peptide vaccines, the direct injection of synthetic peptides or their loading onto DC cells has shown relatively limited efficiency in producing clinical benefits. Instead, the focus has shifted toward using complete proteins or long peptides to activate cellular immunity via cross-presentation. The multi-epitope vaccine designed, as exemplified in this study, falls within the category of long peptides. Consequently, the CTL epitopes within the vaccine can be cross-presented by DCs, leading to the activation of CD8^+^ T cells and the subsequent generation of cellular immunity, as demonstrated in the immune simulations. It is clearly suggestive of the competency of the vaccine construct in eliciting a strong immunization response. However, further *in vitro* and *in vivo* experimentation is necessary to verify the efficacy of the vaccine construct.

## 6 Conclusion

MPX is a highly infectious disease and has spread to 112 countries, yet no safe and specific treatment or vaccine exists against it. In this study, we used structure-guided methods to screen two highly immunogenic proteins from the viral proteome and constructed a novel vaccine candidate against these proteins. It was found to be a promising vaccine design, given the immunological and physiochemical analyses. The molecular docking and MD simulation analyses confirmed that the construct can form stable interactions with immune receptors and, thereby, trigger effective immune responses against MPX. Furthermore, outcomes from immune simulation indicate toward a novel vaccine candidate that can evoke innate as well as antibody-mediated immunity. However, *in vitro* and *in vivo* studies are warranted to assess the efficacy of the candidate in native conditions.

## Data Availability

The raw data supporting the conclusion of this article will be made available by the authors, without undue reservation.

## Abbreviations

MPX, monkeypox; dsDNA, double-stranded DNA; WHO, World Health Organization; FDA, US Food and Drug Administration; DdRp, DNA-dependent RNA polymerase; IEDB, Immune Epitope Database; ACC, auto cross-covariance; MD, molecular dynamics; RMSD, root mean square deviation; RMSF, root mean square fluctuation; Rg, radius of gyration; SASA, solvent-accessible surface area; MM-GBSA, molecular mechanics with generalized Born surface area; PSSM, position-specific scoring matrix; GRAVY, grand average of hydropathy.
