# Supplementary material for: Immunoinformatics and reverse vaccinology approach in designing a novel highly immunogenic multivalent peptide-based vaccine against the human monkeypox virus
Source: Front Mol Biosci. 2023 Nov 22;10:1295817. doi: 10.3389/fmolb.2023.1295817 (PMC10703375; doi:10.3389/fmolb.2023.1295817)
Supplement: Supplementary file 1 [file Table1.DOCX]

**Immunoinformatics and reverse vaccinology approach in designing a novel highly immunogenic multivalent peptide-based vaccine against the Human Monkeypox virus**

**SUPPLEMENTARY MATERIAL**


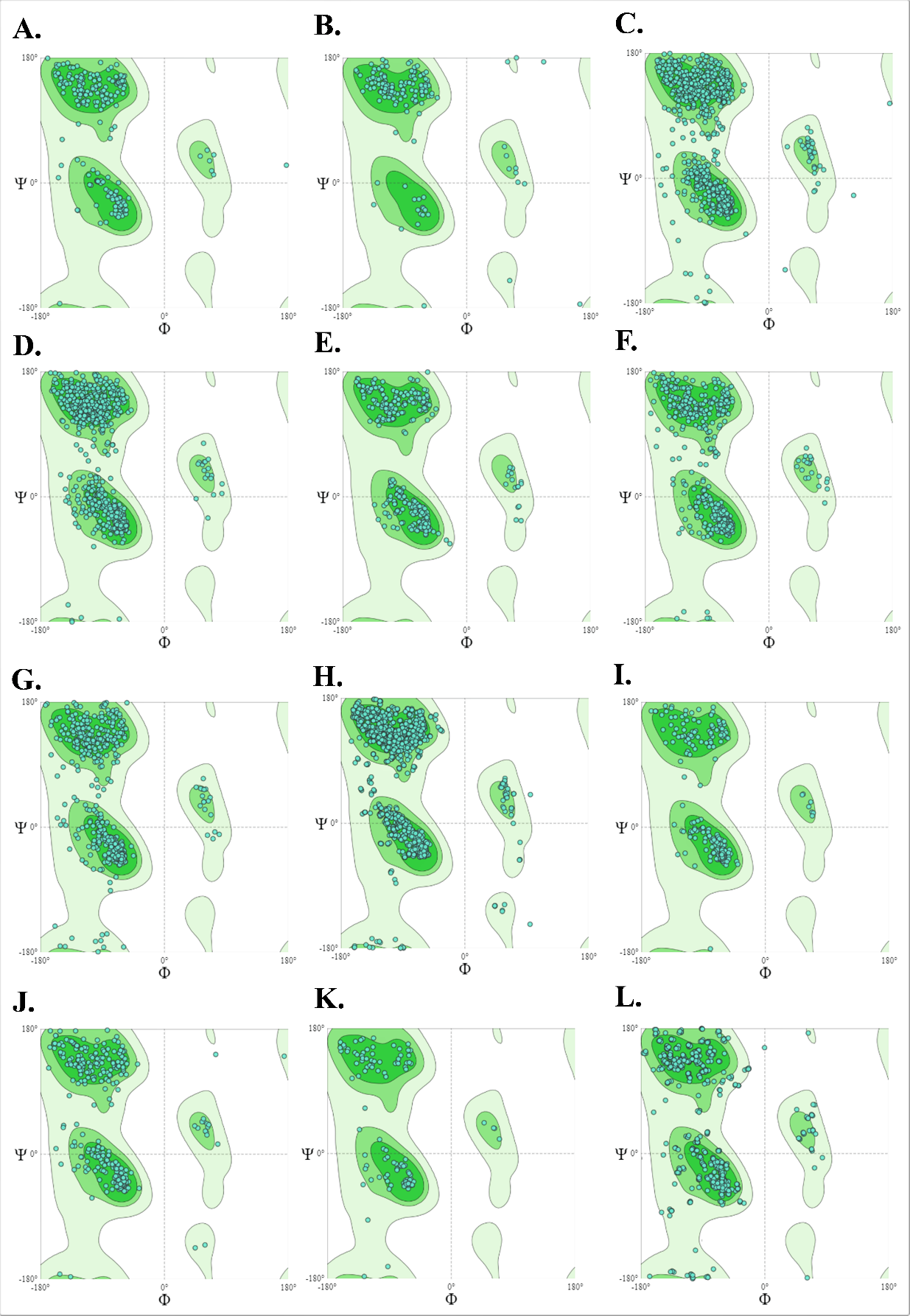


**Supplementary Figure S1.** Shows the Ramachandran plots for A. Cell surface binding protein, B. Complement binding protein, C. DNA-dependent RNA polymerase subunit rpo132, D. E3R, E. E4R, F. E6R, G. E11L, H. E13L, L. H6R, J. Poly A polymerase large subunit, K. Profilin, L. Thymidine kinase.


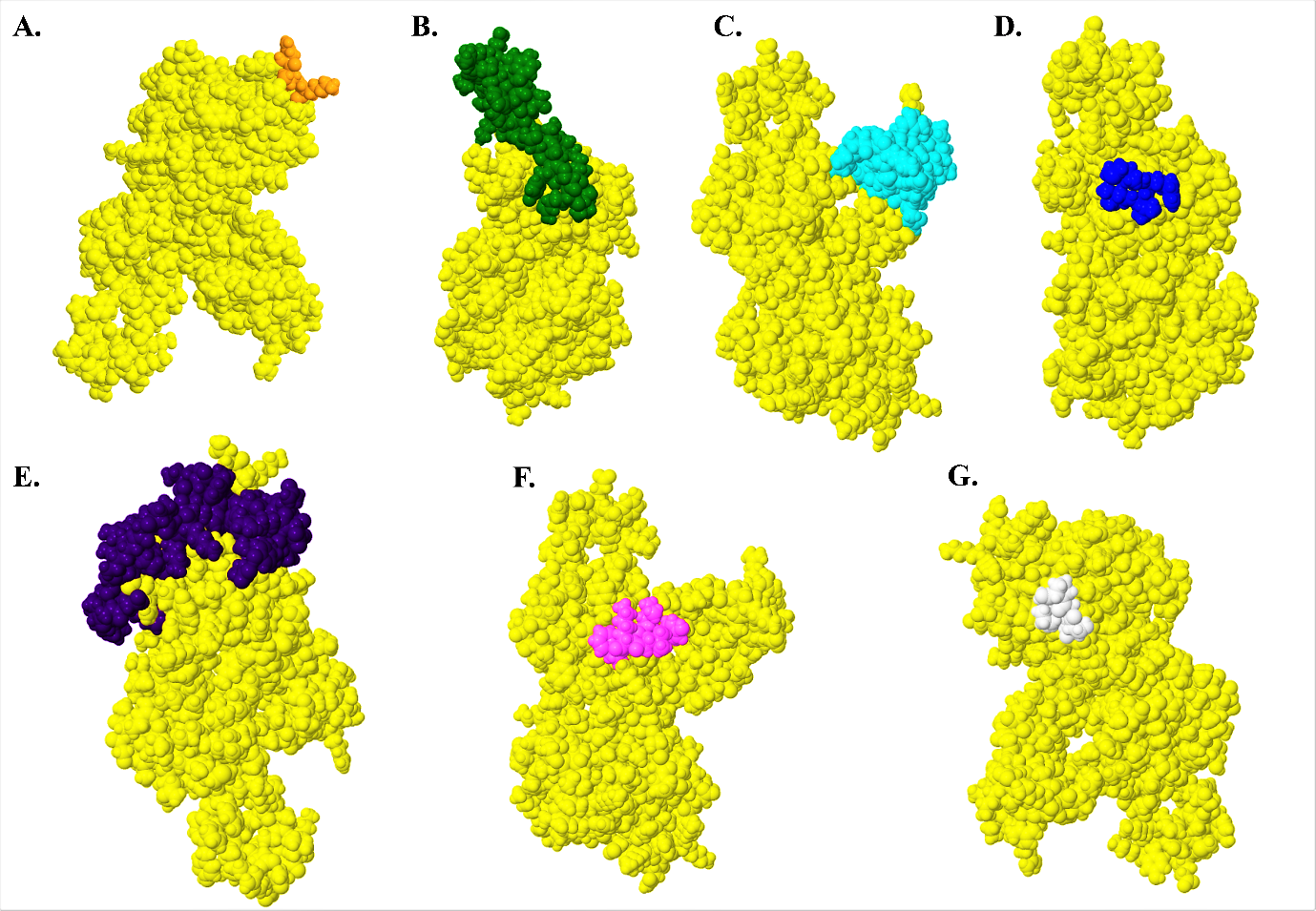


**Supplementary Figure S2.** The 7 different conformational type B-cell epitopes, as extracted by ElliPro.

**Supplementary Table 1.** Interaction of key viral proteins with TLR2 and TLR4

| **Protein** | **ClusPro Dock Scores** | |
| --- | --- | --- |
|  | **TLR2** | **TLR4-MD2** |
| **Cell surface-binding protein** | -640.8 | -794.8 |
| **Thymidine kinase** | -1005.7 | -732.1 |
| **DNA-dependent RNA polymerase subunit rpo132** | -1327.6 | -969.7 |
| **Profilin** | -804.2 | -811.1 |
| **Complement binding Protein** | X | -839.2 |
| **Poly A polymerase large subunit** | -798.2 | -981.6 |
| **E11L** | -872.0 | -825.4 |
| **E13L** | -847.2 | -859.6 |
| **H6R** | -828.6 | -927.7 |
| **E6R** | -1131.8 | -992.9 |
| **E4R** | -724.2 | -736.2 |
| **E3R** | -842.4 | -852.8 |

**Supplementary Table 2.** B-cell epitopes predicted using Bepipred Linear Epitope Prediction 2.0 algorithm

| **Sequence** | **Length** |
| --- | --- |
| **E6R Protein** | |
| VWGVNFRKEYNV | 12 |
| VRQFFYNNSRIKYNDSKLLKMVTSVIKNKEDARNYIDDIVNGHFFVSN | 48 |
| QEMSETYSLPPHPSIVK | 17 |
| DFNDEVTSLNDYTQDELINVLPFDIKKLLYLKFKTKETNR | 40 |
| SYADI | 5 |
| YNSPENDD | 8 |
| KMKSSLED | 8 |
| NGYSEYNGSQGTNPHMINGK | 20 |
| TLFQEQDKELYPNLKINNGVLYGEELVTLNISSK | 34 |
| CYHEMFDKNMYN | 12 |
| SYYEMPDKDLPTIRYHGRKFLDT | 23 |
| IDFGEIISRGKKVIQTLLNERGVNV | 25 |
| IFGNNTGE | 8 |
| NGLSR | 5 |
| MNLFNDEFIAENIFIHSTTSF | 21 |
| IDLFDNHVDSIPTILP | 16 |
| **DNA-dependent RNA polymerase subunit rpo132** | |
| IDLFDNHVDSIPTILP | 16 |
| MNLFNDEFIAENIFIHSTTSF | 21 |
| NGLSR | 5 |
| IFGNNTGE | 8 |
| IDFGEIISRGKKVIQTLLNERGVNV | 25 |
| SYYEMPDKDLPTIRYHGRKFLDT | 23 |
| CYHEMFDKNMYN | 12 |
| TLFQEQDKELYPNLKINNGVLYGEELVTLNISSK | 34 |
| NGYSEYNGSQGTNPHMINGK | 20 |
| KMKSSLED | 8 |
| YNSPENDD | 8 |
| SYADI | 5 |
| DFNDEVTSLNDYTQDELINVLPFDIKKLLYLKFKTKETNR | 40 |
| QEMSETYSLPPHPSIVK | 17 |
| VRQFFYNNSRIKYNDSKLLKMVTSVIKNKEDARNYIDDIVNGHFFVSN | 48 |
| VWGVNFRKEYNV | 12 |

**Supplementary Table 3.** High ranking MHC I epitopes derived by Epitope Prediction and Analysis Tools from IEDB

| **Peptide** | **VaxiJen Score** |
| --- | --- |
| **E6R Protein** | |
| KTFAIVTSK | 0.9752 |
| LLKGRISYY | 0.7869 |
| ALVASRFKK | 0.8187 |
| QILGRSIRK | 0.4510 |
| LPFDIKKLL | 0.7593 |
| KIFNYNMGV | 0.4838 |
| YSLPPHPSI | 0.7789 |
| SLPPHPSIV | 1.0044 |
| FLDTRVVYC | 0.8816 |
| LLYLKFKTK | 2.1463 |
| KTKETNRIY | 0.7054 |
| ITVPFRLSY | 1.5765 |
| TLNISSKFK | 1.4596 |
| TLKGKHFIY | 1.4675 |
| KLQERDYMI | 1.8126 |
| MVTSVIKNK | 0.8288 |
| **DNA-dependent RNA polymerase subunit rpo132** | |
| RLHEILTVK | 0.7280 |
| MPPEVVYLV | 0.7674 |
| VVKPNSFTF | 0.4496 |
| ISITKISSY | 0.9071 |
| VIGGVFINK | 0.7504 |
| KSYDALATF | 0.4840 |
| FQYVSYSNF | 1.5154 |
| YLSRVSLEF | 1.4390 |
| SQYLSRVSL | 0.6930 |
| VTIALMSYK | 1.3999 |
| NLSPLLTKI | 1.1293 |
| RLRHLTQDK | 0.5390 |
| TPPDYSPII | 1.2378 |
| KVGINLVEK | 1.3574 |
| SLSYDMPPEV | 0.6089 |
| FPITIENAL | 0.8573 |
| FARDNQISF | 1.2233 |
| GVFYRPLHF | 0.7274 |

**Supplementary Table 4.** Strong binding MHC II epitopes derived by Epitope Prediction and Analysis Tools from IEDB

| **Peptide** | **VaxiJen Score** |
| --- | --- |
| **E6R Protein** | |
| ILKIFNYNMGVAMNL | 0.4287 |
| RYNNSIFIVDEAHNI | 0.5883 |
| ALLFALVASRFKKVY | 0.5120 |
| IALLFALVASRFKKV | 0.8149 |
| KPKTFAIVTSKMKSS | 0.9890 |
| PKTFAIVTSKMKSSL | 0.7176 |
| **DNA-dependent RNA polymerase subunit rpo132** | |
| QEDGIIIKKQFIQRG | 0.4213 |
| GIIIKKQFIQRGGLD | 0.5344 |
| IIIKKQFIQRGGLDI | 0.4772 |
| EDDFARDNQISFDVS | 0.4577 |
| DDFARDNQISFDVSE | 0.4138 |

**Supplementary Table 5.** Physiochemical characterization of the vaccine peptide as performed by ProtParam

| **Parameters** | **Values** |
| --- | --- |
| **Molecular Weight** | 33.8 kDa |
| **Half-life** | 30 h in mammalian reticulocytes  > 20 h in yeast  > 10 h in *E. coli* |
| **Instability Index** | 22.72 (Stable) |
| **GRAVY** | -0.156 |
| **Aliphatic index** | 94.07 |
| **VaxiJen Score** | 0.8197 (Antigenic) |
| **AllerTOP Score** | -1.024 (Non-Allergen) |
| **VirulentPred Analysis** | Negative (Avirulent) |
